# A Letter from Dr. Clark to Professor Tommasini, in Defence of the Edinburgh School of Medicine

**Published:** 1822-06-01

**Authors:** 


*Lettera di Giacomo Clark, M. D. della Universita' di Edimburgo al ch. Sig. Professore Tommasini, &c. intorno alle sue Osservazioni sulla Scuola Medico-Clinica di Edimburgo, &c.* Pp. 16. Roma, 1822.

When a friend from Italy put into our hands the little pamphlet whose title we have just transcribed, we did not at once recognize our old acquaintance, the author of the "Medical Notes," under the metamorphosis of his Italian name. Many of our readers are aware that, Dr. Clark has resided, for some years past, at Rome, where, we believe, he has obtained among our rich and distinguished countrymen, that annually crowd to the "lone mother of dead empires," the unbounded confidence to which his professional skill and knowledge give him so good a claim. Amid the bustle of practice, and the manifold allurements of so attractive a scene, we are glad to perceive that, the institutions and science of his native country still retain that superior hold on his regard, which their vigorous and philosophical character ought to give them. In the present little work, he stands forward in the face of the whole Italian medical world, and in their own language, as the defender of the English school of medicine, unjustly attacked by one of the most distinguished medical philosophers of the present day in Italy. Whether Dr. *Giacomo* Clark writes in "choice Italian" or not, we fear we are not enough versed in the niceties of that beautiful language to pronounce; but we are sure, that his foreign garb and idiom have detracted nothing from the wonted zeal, simplicity, and good sense, conspicuous in his vernacular productions.

It appears, that Professor Tommasini of Bologna, of whose name we made honourable mention in our last number, visited this country two years since, and on his return, published a report of the state of medicine, and medical education, as observed by him during his residence in Great Britain. Among other things, he gives an account of the Clinical School of Edinburgh; and it is to the exposition and correction of his mis-statements regarding this, that Dr. Clark's letter (addressed to Tommasini himself) is especially devoted. As we are disposed to believe that, the Clinical Institution of Edinburgh is but imperfectly known even in this country, and as we look upon it as the characteristic excellence of that celebrated school, we shall, for the information of our readers, translate Dr. Clark's concise account of it. We shall, however, in the first place, make one or two other extracts from his letter, as affording, at once, a specimen of the degree of knowledge of our medical literature possessed by foreigners, and of the error into which one is likely to fall, in attempting to delineate the full features of a strange land from a short and hasty inspection.

"I cannot help expressing my extreme surprize," says Dr. Clark, "on reading your discourse, to find that you are unacquainted with so many of our most estimable modern authors, a knowledge of whose writings would have enabled you to form a more correct judgment respecting the actual condition of our medical literature and practice. Such are the works of Parr, Saunders, Beddoes, Willan, Currie, Parry, Armstrong *(John)*, Clutterbuck, Johnson, Bateman, Jackson, Cheyne, Marcet, Thomson, Duncan, Philip *(Wilson)* Abernethy, &c. which, (not to mention others) assuredly you could have never read at the time, when you asserted, (p. 19,) that the greater number of English authors 'present little more to us than *individual cases* and *particular histories* of disease, deducing therefrom no general doctrine or principles which can serve as a guide, &c.' Had you been acquainted with our best writers, you would have found that English physicians, although deriving their practical knowledge from the observation of individual cases, (the only legitimate source of practical knowledge,) are sufficiently disposed to generalize their ideas respecting the nature of diseases, and the principles that regulate their treatment. It is indeed true, that they frequently have recourse to individual examples in illustrating and confirming their positions, a practice, not merely advantageous, but rendered often absolutely necessary, by the very imperfection of our art, and, moreover, sanctioned by the authority of the best writers in every country. Our medical literature, it is true, was never *exclusively* devoted to any one theory of disease, but it has certainly been far from being restricted in the manner you mention. On a fuller acquaintance, you would have found that, English medicine could not only boast of being founded on the sure basis of inductive science, but that its philosophic principles afford a faithful guide in practice: most assuredly, you would *not* have found those vacillations of opinion and errors of practice which disfigure the medical history of some other nations, and which we have seen checking the progress of the art in Italy, to this very day" 6.

Dr. C. then adduces the history and fortunes of the Brunonian system in the different European countries, as placing in a clear point of view, the superior discernment of our countrymen.

The following extract from Tommasini's Discourse, seems to contain the main part of his attack on the Edinburgh School.

" Clinical instruction in Edinburgh, is limited to the mere exhibition of what the professor sees in, and prescribes for, a disease,—to the bare history of symptoms and the dictation of remedies; a mode of proceeding, which affords no greater treasure to the student than a collection of cases, and must too frequently lead, in practice, to a servile and perilous imitation. The pupil is neither guided in his studies, nor taught to recognize or define the disease, nor permitted to try his own powers in its treatment under the eye of his teacher and his fellow students. In a word, he neither sees investigated, nor is taught himself to investigate, a disease, in every possible way, as with us in Italy. No explanation is given of the precise circumstances on which the diagnosis, prognosis, and plan of treatment are founded. Nothing is said of the doubts and difficulties which so often leave us uncertain of the truth of our diagnosis, and thereby render our practice inert; nor are the pupils guarded from the risks of a superficial imitation, by having pointed out to them how frequently diseases apparently similar are, in truth, very different, and vice versa. In short, in England no analysis is made of diseases at the bed-side of the patients, and in the presence of the pupils, &c."

The following extracts are from Dr. Clark's reply to those charges of imperfection and inferiority to the Italian schools :—

" A completer examination of the Edinburgh school would have shown you how very different in reality the clinical institutions are from what you state them to be. If you had been present at the *Clinical Lectures* delivered there, (of the existence of which I can hardly believe you to be ignorant, although no mention of them is made in your discourse,) you would have then heard explained the nature of each case, and the manner of "examining it in every possible way, as in Italy," pointed out—the diagnosis and prognosis exposed, the general principles of the cure developed, and the adaptation of these to particular cases determined; in short, you would have heard a full detail of every circumstance which, in the opinion of the professor, could tend to the improvement of the pupil, including (in the fatal cases) a most careful inspection of the body after death." 9.

Dr. C. proceeds to consider the question of the relative merit of clinical lectures delivered apart from, or in presence of, the patients, (the former practice being that of Edinburgh, and the latter that generally adopted in the Italian schools,) and decides, as we think most justly, in favour of the first method. In defending his alma mater from the charge of leading her sons into habits of a servile and perilous imitation of the practice of their teachers, our author strongly retorts the accusation on the very school of Tommasini himself, telling him, with an insinuation sufficiently obvious, that "in Edinburgh no medical professor can enjoy the exclusive opportunity of inculcating a favourite theory or unique practice on the minds of the hearers." "There (he continues) the professor of the practice of medicine is not venerated as the infallible oracle of the healing art, and his peculiar doctrines received as the sole rule of practice; since pupils are obliged to attend the instructions of the other professors also, to accompany them to the hospital, to observe their practice, and to compare it with that of the former."

We now proceed to Dr. C's account of the Edinburgh Clinical School; and, as it is at once extremely concise and correct, we shall translate it for the information of such of our readers (especially the juniors) as have not visited that admirable field for the acquisition of practical knowledge. And we may be allowed here to express our surprise that no clinical wards, on the Edinburgh plan, have hitherto been established in our London hospitals. The want of these is certainly a defect in our metropolitan school, which (very superior as it is to Edinburgh in many other respects) none of its other advantages can compensate. We trust, however, that this defect is not only not irremediable, but that we shall yet see it remedied; and we are well convinced that the individual who shall have the spirit to put such a scheme in effect, will have ample reasons, both public and private, for congratulating himself on its adoption.

"The annual course of clinical instruction at Edinburgh occupies nine months, three professors of the university officiating successively, each three months. Each professor, on his accession to office, selects two of the pupils most advanced in their studies as assistants, (called clerks,) one for the men's ward, the other for the women's. These gentlemen write down the history of each case, on the entry of the patient into the ward, in a very full and distinct manner. At the professor's first visit thereafter, this history is read aloud to him at the patient's bed side, and in the presence of the pupils, when he makes any ulterior examination of the case that may seem to him necessary—calling the attention of the students to any circumstances of the case that may deserve partieular attention, but reserving for the lecture the complete exposition of the nature of the disease. At certain times in the day the journals of the physician's assistants are exhibited in public rooms in the hospital, in order that the students may have an opportunity of transcribing the preliminary histories of the cases into their private journals, to which they afterwards add the daily reports from the vivâ voce dictation of the professor, at the bed-side of the patients.

"Beside the clinical lectures already mentioned for the consideration of the cases individually, each professor, at the termination of his course, gives a general review of the diseases treated by him during the preceding three months—explaining their general character, the effects of the various plans of treatment, and the general results obtained;—instructing, in a word, his pupils to generalize the knowledge obtained from the inspection of the individual cases, and habituating them to the conception and details of practical medicine: the whole forming a course of medical instruction so practically useful and so complete, as can hardly be met with any where else." P. 13.

Among the innumerable blessings of peace, the advancement of the arts and sciences resulting from the free and unrestrained intercourse of nations holds a conspicuous place. This is strikingly illustrated, at this moment, in the case of our own art, by the singular dispersion of our countrymen over the continent of Europe. In France and Italy more especially, there is hardly a great city that does not reckon a resident English physician among its inmates. The practice of these gentlemen, it is true, is in a great measure confined to the colonies of their countrymen, expatriated by fashion, established there; still the influence of their opinions and practice must necessarily extend beyond the immediate sphere of their personal exertions, and thereby affect, more or less, the indigenous science and practice of the respective countries. This will more particularly happen where the interloping practitioners are, like Dr. Clark, of a character and talent that would secure to themselves distinction even at home; and when they do not merely trust to the silent operation of their practice and opinions, displayed in their own small sphere, but, entering the field of foreign literature, boldly challenge the attention of the whole profession to the superior claims of their native country.

